# Impact of vaccine platform and BCG vaccination on antibody responses to COVID-19 vaccination

**DOI:** 10.3389/fimmu.2023.1172851

**Published:** 2023-07-03

**Authors:** Nicole L. Messina, Mariana G. Sperotto, Marco A. M. Puga, Patricia V. da Silva, Roberto D. de Oliveira, Cecilia L. Moore, Laure F. Pittet, Tenaya Jamieson, Margareth Dalcolmo, Glauce dos Santos, Bruno Jardim, Marcus V. G. Lacerda, Nigel Curtis, Julio Croda

**Affiliations:** ^1^ Infectious Diseases Group, Infection and Immunity Theme, Murdoch Children’s Research Institute, Parkville, VIC, Australia; ^2^ Department of Paediatrics, The University of Melbourne, Parkville, VIC, Australia; ^3^ School of Medicine, Federal University of Mato Grosso do Sul, Campo Grande, Mato Grosso do Sul, Brazil; ^4^ Oswaldo Cruz Foundation Mato Grosso do Sul, Campo Grande, Mato Grosso do Sul, Brazil; ^5^ State University of Mato Grosso do Sul, Dourados-Mato Grosso do Sul, Brazil; ^6^ Federal University of Grande Dourados, Dourados, Brazil; ^7^ Clinical Epidemiology and Biostatistics Unit, Murdoch Children’s Research Institute, Parkville, VIC, Australia; ^8^ Infectious Diseases, The Royal Children’s Hospital Melbourne, Parkville, VIC, Australia; ^9^ Helio Fraga Reference Center, Oswaldo Cruz Foundation Ministry of Health, Rio de Janeiro, Rio de Janeiro, Brazil; ^10^ Pontifical Catholic University of Rio de Janeiro, Rio de Janeiro, Rio de Janeiro, Brazil; ^11^ National School of Public Health, Oswaldo Cruz Foundation, Rio de Janeiro, Rio de Janeiro, Brazil; ^12^ Institute of Clinical Research Carlos Borborema, Fundação de Medicina Tropical Doutor Heitor Vieira Dourado, Manaus, Amazonas, Brazil; ^13^ Yale School of Public Health, New Haven, CT, United States

**Keywords:** vaccine, COVID-19, antibody, ChAdOx1-S, CoronaVac, BCG

## Abstract

Multiple factors, including vaccine platform and prior vaccinations, influence vaccine responses. We compared antibody responses to CoronaVac (Sinovac) and ChAdOx1-S (AstraZeneca-Oxford) vaccination in 874 healthcare workers in Brazil. As participants were randomised to BCG vaccination or placebo in the preceding 0-6 months as part of the BCG vaccination to reduce the impact of COVID-19 in healthcare workers (BRACE) trial, we also investigated the influence of recent BCG vaccination on antibody responses to these COVID-19 vaccines. Twenty-eight days after the second dose of each vaccine, ChAdOx1-S induced a stronger anti-spike IgG response than CoronaVac vaccination. Recent BCG vaccination did not impact IgG antibody responses to ChAdOx1-S or CoronaVac.

## 1 Introduction

SARS-CoV-2, the causative pathogen of COVID-19, spreads through the respiratory tract and contact, posing a significant challenge to the healthcare system due to the potential transmission *via* aerosol and droplets (1). The risk of infection is particularly high for healthcare workers, particularly those in urgent care settings, where infection control measures and personal protective equipment may be insufficient (2).

To combat the COVID-19 pandemic, vaccines based on different platforms were developed in parallel. Initially, these included replication deficient adenovirus, mRNA and whole-inactivated virus-based vaccines, with protein sub-unit vaccines being available later in the pandemic. Prior to the COVID-19 pandemic, mRNA and adenovirus-based vaccines had limited use in human and animal models and therefore differences in immune responses to these, and vaccines based on more established platforms (e.g. whole-inactivated, protein subunit) are under investigation.

Due to global demand and limited supply, access to vaccines was limited. Given the increased SARS-CoV-2 exposure in healthcare settings, healthcare workers (along with the elderly) were prioritized for access to COVID-19 vaccinations. In Brazil, the predominant COVID-19 vaccines used for primary vaccination have been ChAdOx1-S (AstraZeneca-Oxford, AZD1222), BNT162b2 (Pfizer-BioNTech), and CoronaVac (Sinovac). ChAdOx1-S, a replication deficient adenovirus-based vaccine, and BNT162b2, an mRNA-based vaccine, induce both humoral and T cell responses to the SARS-CoV-2 spike protein and receptor binding domain (RBD) (3–5). In contrast, CoronaVac, which contains a whole inactivated virus (CN2 strain), primarily provokes a humoral response (6, 7). The primary series schedule for these COVID-19 vaccines is two doses (1, 8), with different intervals depending on the vaccine (9). All three vaccines protect against COVID-19, however reported efficacy is variable with 91-95% reported for BNT162b2 (10, 11), 70-81% for ChAdOx1-S (6, 10, 12) and 50-67% for CoronaVac (6, 10, 11).

Several factors influence the magnitude and persistence of immune responses to COVID-19 vaccines including prior SARS-CoV-2 infection, age and sex (13–16). The immunomodulatory effects of the Bacillus Calmette-Guérin (BCG) vaccine include altering *in vitro* immune responses to SARS-CoV-2 and antibody response to unrelated vaccines, therefore BCG vaccination may also influence immune responses to COVID-19 vaccines (2, 17–23). This study aimed to compare ChAdOx1-S (AstraZeneca-Oxford) and CoronaVac (Sinovac) antibody responses and evaluate the impact of BCG vaccination on the vaccine responses.

## 2 Methods

### 2.1 Participants and sample collection

Participants were recruited as part of the ‘BRACE COVID-19-specific’ (BCOS) sub-study of the *BCG vaccination to reduce the impact of COVID-19 in healthcare workers* (BRACE) trial (24) (human research ethics committee approvals: Royal Children’s Hospital (RCH) approval no. 62586;

Brazilian National Commission of Ethics in Research (CONEP): approval no. 4.572.593). BRACE trial participants in Brazil (recruited from BRACE trial sites in Mato Grosso do Sul, Rio de Janeiro and Amazonas) were eligible for inclusion if: (i) they consented to be contacted for future ethically approved projects; (ii) had a post-vaccination serum sample taken 28 (± 2 days) after the first or second dose of a COVID-19 vaccine.

### 2.2 Vaccination and randomisation

Participants received COVID-19 vaccinations through the Brazilian healthcare system and as per guidelines from the Brazilian Ministry of Health, no treatment was provided for symptoms resulting from COVID-19 vaccination.

Prior to consent to this sub-study and COVID-19 vaccination, participants had been randomised at a 1:1 ratio to receive a 0.1 ml intradermal injection with BCG-Denmark (AJ Vaccines – Batch: 1198019D/119053A) or placebo (0.9% saline solution) as part of the BRACE trial. Eighteen participants consented to this sub-study received BNT162b2, and due to this low number were excluded from statistical analyses.

### 2.3 Sample collection

Serum samples were collected between 19th March 2021 and 10th September 2021. Peripheral blood was collected in serum tubes (BD Vacutainer SS II Advanced, United Kingdom, Cat #456010) at study visits 28 (± 2) days following the first (ChAdOx1-S, BNT162b2) or second (ChAdOx1-S, BNT162b2, CoronaVac) COVID-19 vaccine dose. The recommended 21-day window between the first and second dose of CoronaVac precluded assessment of antibody responses 28 days after the first dose of CoronaVac. Peripheral blood collected in serum tubes was centrifuged and serum samples were stored at -80°C prior to testing.

For plasma samples, peripheral blood was collected into lithium heparin tubes (Greiner BioOne – Austria, Cat #455084), at baseline, 3 months or 6-months after randomisation as part of the BRACE trial. Peripheral blood was centrifuged, and plasma samples were stored at -80°C. The participant’s most recent plasma sample available prior to COVID-19 vaccination was used in this study.

### 2.4 Antibody measurement

Plasma and serum samples were tested for antibodies against the spike receptor-binding domain of SARS-CoV-2 at Unidade de Apoio ao Diagnóstico do COVID-19 (UNADIG-RJ) using the SARS-CoV-2 IgG (Abbott, United States of America) chemiluminescent microparticle immunoassays (CMIA). Seroconversion was defined as a positive anti-SARS-CoV-2 spike antibody result in the post-vaccination sample following a negative anti-SARS-CoV-2 spike antibody result in the pre-vaccination sample. A positive anti-SARS-CoV-2 spike antibody result was defined as per the manufacturer’s defined cut-off. Researchers involved in sample processing, selection and testing were blinded to the participants randomisation group and were unaware of which COVID-19 vaccine they had received.

### 2.5 Interferon gamma release assay

Peripheral blood collected on the day of randomisation in the BRACE trial was used for IGRA to identify *Mycobacterium tuberculosis* infection. The QuantiFERON-TB Gold Plus (QFT-Plus) assay (Qiagen, Hilden, Germany) was done as per manufacturers instructions. Results of IGRA testing were assessed according to the manufacturer’s criteria, using Qiagen software version 2.71.2.

### 2.6 Statistical analysis

For seroconversion analysis, 850 (99.3%) participants had a pre-vaccination plasma sample available for analysis. Differences in proportion were determined using the z-test for independent proportions.

For assessment of antibody concentrations, values below the lower limit of detection/extrapolation were assigned a value of half of the lowest detected/extrapolated value, values above the upper limit of detection/extrapolation were assigned a value of 1.5 times the highest detected/extrapolated value. Differences between COVID-19 vaccines were assessed using linear regression of log-transformed antibody data adjusted for region (Mato Grosso do Sul/Rio de Janeiro/Amazonas), age (<40 years; 40 to 59 years; ≥60 years), sex (male/female), presence of cardiovascular disease, diabetes, chronic respiratory disease, workplace COVID-19 direct contact at baseline (yes/no), SARS-CoV-2 PCR result at baseline (detected/not detected/not performed). Differences between randomisation groups were assessed using linear regression of log-transformed antibody data. Sensitivity analysis for differences between BCG- and placebo-vaccinated participants was done adjusting for time between most recent COVID-19 vaccination and blood collection (continuous), age (<40 years; 40 to 59 years; ≥60 years), sex (male/female) and region (Mato Grosso do Sul; Rio de Janeiro; Amazonas). Data analysis was done using Stata version 17.0 (StataCorp LLC, USA)

The authors declare that the data supporting the findings of this study are available within the paper and its supplementary information files.

## 3 Results

**Figure 1:**
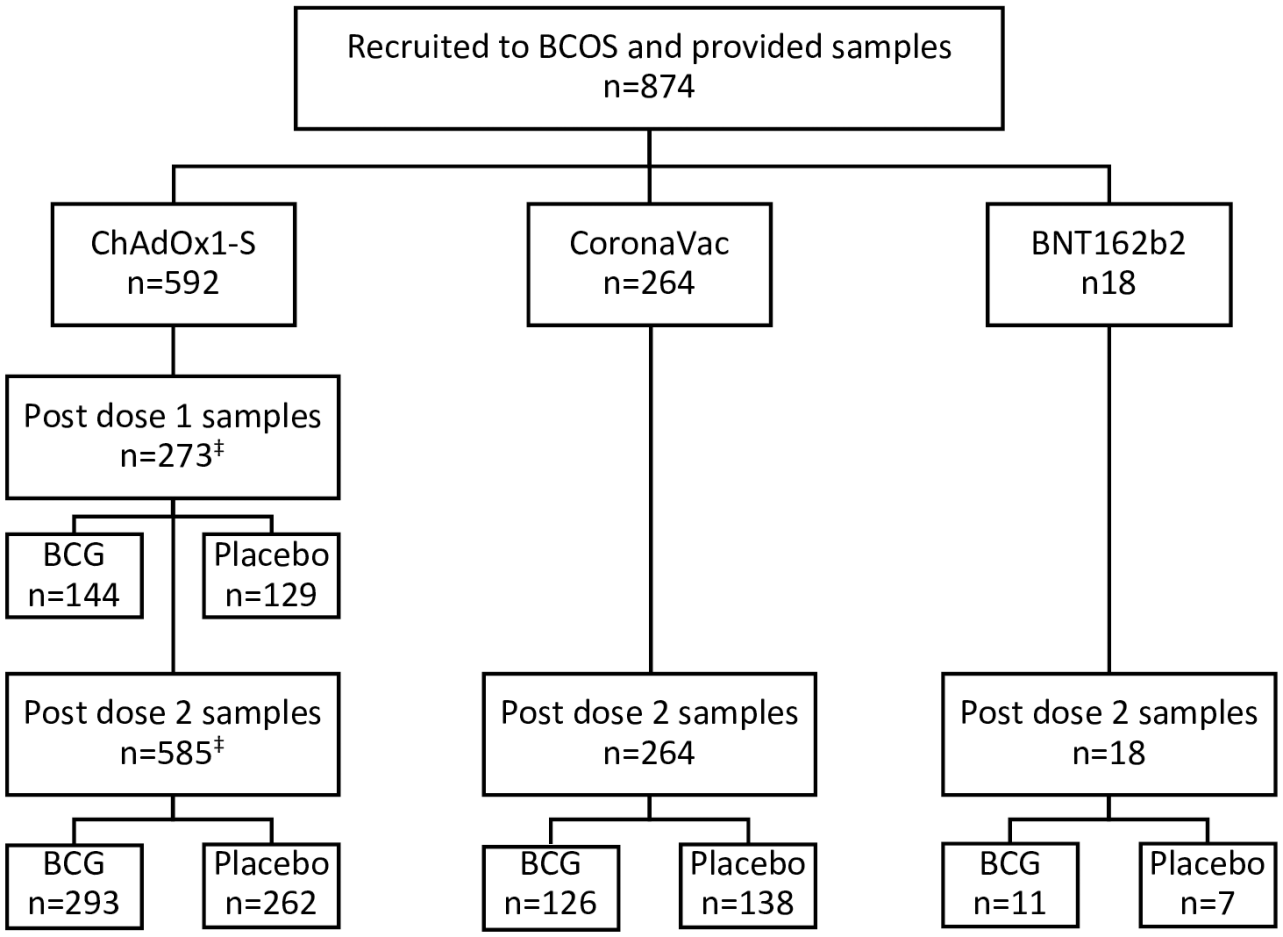
Participant flow chart. Participants in the BRACE COVID-19-Specfic vaccine (BCOS) sub-study were a subset of participants from the BRACE trial. BRACE trial participants in Brazil who received (or were due to receive) two doses of COVID-19-specific vaccines prior to August 9^th^ 2021 and provided at least 1 BCOS-post-vaccination serum sample were eligible for inclusion. Participants were randomised to BCG or placebo vaccination in the BRACE trial a mean of 80 (standard deviation 41 days) prior to their first COVID-19 vaccination. ^‡^266 participants were included for dose 1 and dose 2 of ChAdOx1-S.

Of participants who consented to this sub-study, 874 completed COVID-19 vaccination courses before 9th August 2021 and provided post-vaccination blood samples (**Figure 1**). Blood samples were taken a median of 28 (IQR 27-29) days after the first (ChAdOx1-S, BNT162b2) and 28 (IQR 27-30) days after the second COVID-19 (ChAdOx1-S, BNT162b2 or CoronaVac) vaccination. The demographics of participants are detailed in **Supplementary Tables 1, 2**. The mean time between randomisation in the BRACE trial and the first and second doses of SARS-CoV-2 vaccines was 80 (standard deviation, SD, 25) days and 145 (SD 26) days, respectively (**Supplementary Table 2**). Only 4% of individuals in the BCG arm and 2% in the placebo arm had another vaccination between randomisation and the first dose of a COVID-19 vaccine. For both randomisation arms, 18% of participants received another vaccination between randomisation and the second dose of a COVID-19 vaccine. SARS-CoV-2 infection at the time of randomisation (as determined by respiratory swab SARS-CoV-2 PCR test) and *M. tuberculosis* infection (determined by IGRA), was similar between randomisation arms and between ChAdOx1-S and CoronaVac recipients (**Supplementary Tables 1, 2**).

### 3.1 Level of anti-spike IgG between COVID-19 vaccines

The concentration of anti-spike IgG antibodies induced by a single dose of ChAdOx1-S was similar to that induced by two doses of CoronaVac (adjusted geometric mean ratio (aGRM) 0.99, 95% CI 0.76-1.30) (**Table 1**). Anti-spike IgG antibody levels were more than two times greater following two doses of ChAdOx1-S than two doses of CoronaVac (aGMR 2.58, 95% CI 2.19-3.03).

**Table 1:** Comparison of anti-spike IgG antibody concentration following ChAdOx1-S or CoronaVac vaccination among healthcare workers in Brazil.

	ChAdOx1-S (n=592)	CoronaVac (n=264)	aGMR* (95% CI)	P-value*
	1^st^ dose	2^nd^ dose		
No. of available samples	273	264	0.99 (0.76, 1.30)	0.97
Geometric mean (95% CI)	970.4 (789.6, 1192.6)	972.3 (869.4, 1087.4)		
	2^nd^ dose	2^nd^ dose		
No. of available samples	585	264	2.58 (2.19, 3.03)	<0.001
Geometric mean (95% CI)	2439.3 (2225.4, 2673.9)	972.30 (869.4, 1087.4)		

*Adjusted for region, age, sex, presence of cardiovascular disease, diabetes, chronic respiratory disease, workplace COVID-19 direct contact at baseline, SARS-CoV-2 PCR result at baseline

95% CI, 95% confidence interval; aGMR, adjusted geometric mean ratio; Ig, immunoglobulin; No., number

### 3.2 Impact of recent BCG vaccination on level of anti-spike IgG

Prior to receiving COVID-19 vaccinations, participants were randomised to receive vaccination with BCG-Denmark (n=435) or saline placebo (n=439) as part of the BRACE trial (24). Amongst ChAdOx1-S and CoronaVac recipients, there was no difference in the anti-spike IgG seroconversion rate or geometric mean anti-spike IgG responses between BCG-vaccinated and placebo-vaccinated participants (**Table 2**; **Supplementary Table 3, 4**). Adjustment for time between the most recent COVID-19 vaccination and post-vaccination blood collection, age, sex, and region did not impact these findings (**Supplementary Table 4**).

**Table 2:** Comparison of anti-spike IgG antibody concentration following ChAdOx1-S or CoronaVac vaccination among healthcare workers in Brazil by BCG randomisation group.

	Placebo (N=439)	BCG (N=435)	GMR (95% CI)	P-value
1st dose of ChAdOx1-S				
No. of available samples	129	144	1.01 (0.67, 1.52)	0.97
Geometric mean (95% CI)	965.8 (718.9, 1297.4)	974.5 (730.1, 1300.8)		
2nd dose of ChAdOx1-S				
No. of available samples	292	293	0.92 (0.77, 1.11)	0.38
Geometric mean (95% CI)	2540.8 (2254.3, 2863.8)	2342.2 (2037.7, 2692.2)		
2nd dose of CoronaVac				
No. of available samples	138	126	1.03 (0.83, 1.30)	0.76
Geometric mean (95% CI)	956.5 (819.7, 1116.2)	989.9 (841.1, 1165.1)		

95% CI, 95% confidence interval; GMR, geometric mean ratio; Ig, immunoglobulin; No., number.

## 4 Discussion

### 4.1 Effect of COVID-19 vaccine platform

Our finding that ChAdOx1-S induced higher anti-spike IgG levels compared to CoronaVac may be explained by the fact that this vaccine is an attenuated virus vector, which activates a broader range of T cells and induces a stronger cytokine production (3), ultimately associated with a higher efficacy (13).

COVID-19 vaccines are a vital tool in protecting against the disease and particularly severe forms. Randomised control trials (RCTs) of ChAdOx1-S have reported an overall protective efficacy of 81% (25) against symptomatic COVID-19, with lower protection associated with COVID-19 caused by the variant of concerns (26, 27) Previous studies have shown that levels of neutralizing antibodies correlate with vaccine efficacy (28).

Phase III RCTs of CoronaVac have reported protective efficacy varying between 51% in Brazil (29) and 84% in Turkey (30). More recently, a national cohort study on over 10 million persons in Chile reported an efficacy of 65.9% (31). The difference we observed in the anti-spike IgG responses is consistent with the observed higher protective efficacy of ChAdOx1-S against symptomatic COVID-19 than CoronaVac, and the observed magnitude is consistent with reported differences in neutralizing antibodies between these vaccines (32).

Used in an emergency, CoronaVac was approved for vaccination in healthcare workers due to direct contact with COVID-19 patients, leading to higher SARS-CoV-2 exposure than the general population. In a recent cohort study of healthcare workers in Brazil, seropositivity for anti-spike IgG was 99.8% 30 days after the second dose of CoronaVac, and decreased to 97.9% six months after the second doses was 97.0-100% (33). Antibody positivity as well as antibody titre were higher in females compared to males, with this sex effect maintained across a range of ages.

Prior SARS-CoV-2 infection is associated with stronger responses to COVID-19 vaccines (14, 15, 34). In our study, workplace and household exposure, as well as study region were evenly distributed between the BCG and placebo groups but were unbalanced between participants with samples 28 days after the second dose of ChAdOx1-S and CoronaVac. Due to differences in SARS-CoV-2 prevalence across the regions over time, the risk of SARS-CoV-2 infections among participants will have varied between regions (35, 36). Higher workplace patient- and household-COVID-19 contact for participants who received CoronaVac, might also have influenced our findings.

### 4.2 Effect of recent BCG vaccination

Due to its beneficial off-target effects in protecting against unrelated infections (20, 37–39), BCG vaccination has been investigated for its potential to protect against COVID-19. Ecological studies have reported variable effects of BCG with some studies showing that countries with BCG vaccination programs had fewer COVID-19 cases, while others found effect of BCG vaccination (19). RCTs of BCG vaccination to protect against COVID-19 are ongoing, however thus far results have also been variable (40). One such study, the Dutch BCG-Corona study, found that BCG-Denmark vaccination did not reduce COVID-19 incidence, duration or severity in healthcare workers (2). This study did however report a transient effect of BCG vaccination on antibody responses to SARS-CoV-2. In the first three months after randomisation, participants in the BCG-vaccinated group had higher rates of seroconversion and anti-spike antibody titres compared to the placebo group, although this effect was not maintained at later time points (2). It remains unclear whether this was a direct effect of BCG vaccination on humoral responses to SARS-CoV-2 or an indirect effect such as being the result of differences in SARS-CoV-2 clearance and viral load.

Our findings, that recent BCG vaccination did not alter IgG responses to CoronaVac or ChAdOx1-S, are consistent with a study that reported the lack of an increase in B cell responses to SARS-CoV-2 *in vitro* in BCG-vaccinated participants compared to placebo controls (22). Similarly, a Polish phase III RCT also found that mycobacterial exposure (positive tuberculin skin test or those BCG-Moreau vaccination) did not affect anti-spike IgG responses to BNT162b2 compared to the placebo (41). BCG-induced trained immunity occurs rapidly and persists for at least 1 year following neonatal BCG vaccination (42–45), therefore it is unlikely that the lack of an observed effect of BCG is due to waning of its immunomodulatory effects in the months between randomisation and COVID-19 vaccinations. Although BCG vaccination did not impact antibody responses in this study, prior BCG vaccination may alter innate or T cell responses to COVID-19 vaccines and may be a viable adjuvant for novel COVID-19 vaccines (20, 37, 46, 47).

Prior mycobacterial exposure among placebo-vaccinated participants may have reduced the observed effects of recent BCG vaccination in our study. In animal models, exposure to environmental mycobacteria, such as *Mycobacterium avium*, can reduce BCG-induced protective immunity against TB (48, 49). However, these effects have been shown to be variable, depending on factors such as mycobacterium species and route of exposure (50). In human trials, stringent tuberculin testing and younger age at BCG vaccination, both of which reduce the likelihood of prior *M. tuberculosis* and environmental mycobacterial exposure, are also associated with greater BCG efficacy (51). The effects of environmental mycobacterial exposure on the off-target effects of BCG vaccination are unknown.

Being based in a high-tuberculosis (TB) prevalence setting, most study participants had received prior BCG vaccination, over 95% of which was more than 5 years prior, likely as part of routine infant BCG vaccination programmes. Infants whose mothers had also been BCG-vaccinated in the past have been reported to have stronger clinical and immunological off-target effects of BCG vaccination (44, 52, 53). BCG revaccination later in life may provide limited additional effect (54–56). Therefore, the lack of an observed boosting effect of recent BCG vaccination may be attributable, at least in part, to prior mycobacterial exposure.

### 4.3 Limitations

The study’s limitations include that participants were not randomised to the different COVID-19 vaccines, and there were some differences in baseline demographics between the groups. However, factors previously associated with antibody responses to COVID-19 vaccines, such as sex, age and comorbidities (13–16), were evenly distributed between the groups and we tried to account for the impact of any differences in demographics in the analysis. As the study was done in a high COVID-19 prevalence setting and pre-vaccination samples were taken up to six months before the post-COVID-19 vaccination samples, seroconversion and vaccine-induced antibody titres may have been overestimated due to COVID-19 infections between pre and post-vaccination sample collections. This may be due to measurement of SARS-CoV-2-induced antibody responses directly, as well as the increased antibody responses to COVID-19 vaccine among previously SARS-CoV-2-infected participants (14, 15, 34). Other factors that can influence antibody responses to COVID-19 vaccines, including participant health, self-administration of antipyretics, immune-stimulants and immune suppressants by participants was not recorded and therefore their potential impact on the effects of BCG or COVID-19 vaccine-induced antibody responses could not be determined (57, 58). As a sub-study of an RCT in healthcare workers, the participants included were predominantly females under 60 years of age. As sex and age are factors that influence vaccine responses (13–16), it is plausible that antibody titres in response to ChAdOx1-S and CoronaVac, and the effects of BCG may be different among different subgroups.

### 4.4 Conclusions

In conclusion, in healthcare workers in Brazil, vaccination with the adenovirus-based ChAdOx1-S vaccine induced greater anti-spike IgG responses than whole-inactivated virus CoronaVac vaccine, and recent BCG vaccination had no effect on anti-spike antibody vaccine responses.

## Data availability statement

The original contributions presented in the study are included in the article/**Supplementary Material**. Further inquiries can be directed to the corresponding author.

## Ethics statement

The studies involving human participants were reviewed and approved by Royal Children’s Hospital (RCH) (no. 62586) and Brazilian National Research Ethics Committee (no. CAAE 34101620.5.1001.0021). The patients/participants provided their written informed consent to participate in this study.

## Author contributions

NM, NC, and JC conceived and designed the project. NM and NC acquired funding. NM, JC, MP, MD, TJ, PS, RO, MG, and BJ executed the study. CM, NM, NC, and LP developed the statistical analysis plan. CM completed the statistical analysis. NM, NC, CM, JC, MP contributed to interpretation of the data. MS, NM, and NC contributed to the writing of the manuscript. All authors contributed to the article and approved the submitted version.

## References

[1] Gordon C Porteous D Unsworth J. COVID-19 vaccines and vaccine administration. Br J Nurs. (2021) 30(6):344–9. doi: 10.12968/bjon.2021.30.6.344 33769885

[2] Claus J Ten Doesschate T Gumbs C van Werkhoven CH van der Vaart TW Janssen AB. BCG Vaccination of health care workers does not reduce SARS-CoV-2 infections nor infection severity or duration: a randomized placebo-controlled trial. mBio (2023) 14 (2):e0035623. doi: 10.1128/mbio.00356-23 36976004 PMC10128007

[3] Ewer KJ Barrett JR Belij-Rammerstorfer S Sharpe H Makinson R Morter R. T Cell and antibody responses induced by a single dose of ChAdOx1 nCoV-19 (AZD1222) vaccine in a phase 1/2 clinical trial. Nat Med (2021) 27(2):270–8. doi: 10.1038/s41591-020-01194-5 33335323

[4] Ramasamy MN Minassian AM Ewer KJ Flaxman AL Folegatti PM Owens DR. Safety and immunogenicity of ChAdOx1 nCoV-19 vaccine administered in a prime-boost regimen in young and old adults (COV002): a single-blind, randomised, controlled, phase 2/3 trial. Lancet. (2021) 396(10267):1979–93. doi: 10.1016/S0140-6736(20)32466-1 PMC7674972 33220855

[5] Sahin U Muik A Derhovanessian E Vogler I Kranz LM Vormehr M. COVID-19 vaccine BNT162b1 elicits human antibody and T(H)1 T cell responses. Nature. (2020) 586(7830):594–9. doi: 10.1038/s41586-020-2814-7 32998157

[6] Hadj Hassine I. Covid-19 vaccines and variants of concern: a review. Rev Med Virol (2022) 32(4):e2313. doi: 10.1002/rmv.2313 34755408 PMC8646685

[7] Zhang Y Zeng G Pan H Li C Hu Y Chu K. Safety, tolerability, and immunogenicity of an inactivated SARS-CoV-2 vaccine in healthy adults aged 18-59 years: a randomised, double-blind, placebo-controlled, phase 1/2 clinical trial. Lancet Infect Dis (2021) 21(2):181–92. doi: 10.1016/S1473-3099(20)30843-4 PMC7832443 33217362

[8] Hogan MJ Pardi N. mRNA vaccines in the COVID-19 pandemic and beyond. Annu Rev Med (2022) 73:17–39. doi: 10.1146/annurev-med-042420-112725 34669432

[9] Tregoning JS Flight KE Higham SL Wang Z Pierce BF. Progress of the COVID-19 vaccine effort: viruses, vaccines and variants versus efficacy, effectiveness and escape. Nat Rev Immunol (2021) 21(10):626–36. doi: 10.1038/s41577-021-00592-1 PMC8351583 34373623

[10] Carneiro DC Sousa JD Monteiro-Cunha JP. The COVID-19 vaccine development: a pandemic paradigm. Virus Res (2021) 301:198454. doi: 10.1016/j.virusres.2021.198454 34015363 PMC8127526

[11] Zheng C Shao W Chen X Zhang B Wang G Zhang W. Real-world effectiveness of COVID-19 vaccines: a literature review and meta-analysis. Int J Infect Dis (2022) 114:252–60. doi: 10.1016/j.ijid.2021.11.009 PMC8595975 34800687

[12] Voysey M Clemens SAC Madhi SA Weckx LY Folegatti PM Aley PK. Safety and efficacy of the ChAdOx1 nCoV-19 vaccine (AZD1222) against SARS-CoV-2: an interim analysis of four randomised controlled trials in Brazil, south Africa, and the UK. Lancet. (2021) 397(10269):99–111. doi: 10.1016/S0140-6736(20)32661-1 33306989 PMC7723445

[13] Barin B Kasap U Selcuk F Volkan E Uluckan O. Comparison of SARS-CoV-2 anti-spike receptor binding domain IgG antibody responses after CoronaVac, BNT162b2, ChAdOx1 COVID-19 vaccines, and a single booster dose: a prospective, longitudinal population-based study. Lancet Microbe (2022) 3(4):e274–e83. doi: 10.1016/S2666-5247(21)00305-0 PMC8828370 35165669

[14] Ebinger JE Joung S Liu Y Wu M Weber B Claggett B. Demographic and clinical characteristics associated with variations in antibody response to BNT162b2 COVID-19 vaccination among healthcare workers at an academic medical centre: a longitudinal cohort analysis. BMJ Open (2022) 12(5):e059994. doi: 10.1136/bmjopen-2021-059994 PMC9130668 35613792

[15] Romero-Ibarguengoitia ME Gonzalez-Cantu A Pozzi C Levi R Mollura M Sarti R. Analysis of immunization time, amplitude, and adverse events of seven different vaccines against SARS-CoV-2 across four different countries. Front Immunol (2022) 13:894277. doi: 10.3389/fimmu.2022.894277 35967368 PMC9367469

[16] Uwamino Y Kurafuji T Sato Y Tomita Y Shibata A Tanabe A. Young age, female sex, and presence of systemic adverse reactions are associated with high post-vaccination antibody titer after two doses of BNT162b2 mRNA SARS-CoV-2 vaccination: an observational study of 646 Japanese healthcare workers and university staff. Vaccine. (2022) 40(7):1019–25. doi: 10.1016/j.vaccine.2022.01.002 PMC8739021 35033389

[17] Netea MG van der Meer JW van Crevel R. BCG Vaccination in health care providers and the protection against COVID-19. J Clin Invest. (2021) 131(2):e145545. doi: 10.1172/JCI145545 33306484 PMC7810495

[18] Rivas MN Ebinger JE Wu M Sun N Braun J Sobhani K. BCG Vaccination history associates with decreased SARS-CoV-2 seroprevalence across a diverse cohort of health care workers. J Clin Invest (2021) 131(2):e145157. doi: 10.1172/JCI145157 PMC7810479 33211672

[19] Gonzalez-Perez M Sanchez-Tarjuelo R Shor B Nistal-Villan E Ochando J. The BCG vaccine for COVID-19: first verdict and future directions. Front Immunol (2021) 12:632478. doi: 10.3389/fimmu.2021.632478 33763077 PMC7982405

[20] Messina NL Zimmermann P Curtis N. The impact of vaccines on heterologous adaptive immunity. Clin Microbiol Infect (2019) 25(12):1484–93. doi: 10.1016/j.cmi.2019.02.016 30797062

[21] Zimmermann P Curtis N. The influence of BCG on vaccine responses - a systematic review. Expert Rev Vaccines (2018) 17(6):547–54. doi: 10.1080/14760584.2018.1483727 29882438

[22] Messina NL Germano S McElroy R Rudraraju R Bonnici R Pittet LF. Off-target effects of bacillus calmette-guerin vaccination on immune responses to SARS-CoV-2: implications for protection against severe COVID-19. Clin Transl Immunol (2022) 11(4):e1387. doi: 10.1002/cti2.1387 PMC9028103 35573165

[23] Moorlag S Taks E Ten Doesschate T van der Vaart TW Janssen AB Muller L. Efficacy of BCG vaccination against respiratory tract infections in older adults during the coronavirus disease 2019 pandemic. Clin Infect Dis (2022) 75(1):e938–e46. doi: 10.1093/cid/ciac182 PMC8903481 35247264

[24] Pittet LF Messina NL Gardiner K Orsini F Abruzzo V Bannister S. BCG Vaccination to reduce the impact of COVID-19 in healthcare workers: protocol for a randomised controlled trial (BRACE trial). BMJ Open (2021) 11(10):e052101. doi: 10.1136/bmjopen-2021-052101 PMC8557250 34711598

[25] Falsey AR Sobieszczyk ME Hirsch I Sproule S Robb ML Corey L. Phase 3 safety and efficacy of AZD1222 (ChAdOx1 nCoV-19) covid-19 vaccine. N Engl J Med (2021) 385(25):2348–60. doi: 10.1056/NEJMoa2105290 PMC8522798 34587382

[26] Madhi SA Baillie V Cutland CL Voysey M Koen AL Fairlie L. Efficacy of the ChAdOx1 nCoV-19 covid-19 vaccine against the B.1.351 variant. N Engl J Med (2021) 384(20):1885–98. doi: 10.1056/NEJMoa2102214 PMC7993410 33725432

[27] Emary KRW Golubchik T Aley PK Ariani CV Angus B Bibi S. Efficacy of ChAdOx1 nCoV-19 (AZD1222) vaccine against SARS-CoV-2 variant of concern 202012/01 (B.1.1.7): an exploratory analysis of a randomised controlled trial. Lancet (2021) 397(10282):1351–62. doi: 10.1016/S0140-6736(21)00628-0 PMC8009612 33798499

[28] Gilbert PB Donis RO Koup RA Fong Y Plotkin SA Follmann D. A covid-19 milestone attained - a correlate of protection for vaccines. N Engl J Med (2022) 387(24):2203–6. doi: 10.1056/NEJMp2211314 36507702

[29] Hitchings MDT Ranzani OT Torres MSS de Oliveira SB Almiron M Said R. Effectiveness of CoronaVac among healthcare workers in the setting of high SARS-CoV-2 gamma variant transmission in manaus, Brazil: a test-negative case-control study. Lancet Reg Health Am (2021) 1:100025. doi: 10.1016/j.lana.2021.100025 34386791 PMC8310555

[30] Tanriover MD Doganay HL Akova M Guner HR Azap A Akhan S. Efficacy and safety of an inactivated whole-virion SARS-CoV-2 vaccine (CoronaVac): interim results of a double-blind, randomised, placebo-controlled, phase 3 trial in Turkey. Lancet. (2021) 398(10296):213–22. doi: 10.1016/S0140-6736(21)01429-X PMC8266301 34246358

[31] Jara A Undurraga EA Gonzalez C Paredes F Fontecilla T Jara G. Effectiveness of an inactivated SARS-CoV-2 vaccine in Chile. N Engl J Med (2021) 385(10):875–84. doi: 10.1056/NEJMoa2107715 PMC8279092 34233097

[32] Khoury DS Cromer D Reynaldi A Schlub TE Wheatley AK Juno JA. Neutralizing antibody levels are highly predictive of immune protection from symptomatic SARS-CoV-2 infection. Nat Med (2021) 27(7):1205–11. doi: 10.1038/s41591-021-01377-8 34002089

[33] Fonseca MHG de Souza TFG de Carvalho Araujo FM de Andrade LOM. Dynamics of antibody response to CoronaVac vaccine. J Med Virol (2022) 94(5):2139–48. doi: 10.1002/jmv.27604 PMC9015561 35060174

[34] Anichini G Terrosi C Gandolfo C Gori Savellini G Fabrizi S Miceli GB. SARS-CoV-2 antibody response in persons with past natural infection. N Engl J Med (2021) 385(1):90–2. doi: 10.1056/NEJMc2103825 PMC8063888 33852796

[35] Castro MC Kim S Barberia L Ribeiro AF Gurzenda S Ribeiro KB. Spatiotemporal pattern of COVID-19 spread in Brazil. Science. (2021) 372(6544):821–6. doi: 10.1126/science.abh1558 33853971

[36] Giovanetti M Slavov SN Fonseca V Wilkinson E Tegally H Patane JSL. Genomic epidemiology of the SARS-CoV-2 epidemic in Brazil. Nat Microbiol (2022) 7(9):1490–500. doi: 10.1038/s41564-022-01191-z PMC9417986 35982313

[37] Arts RJW Moorlag S Novakovic B Li Y Wang SY Oosting M. BCG Vaccination protects against experimental viral infection in humans through the induction of cytokines associated with trained immunity. Cell Host Microbe (2018) 23(1):89–100 e5. doi: 10.1016/j.chom.2017.12.010 29324233

[38] Goodridge HS Ahmed SS Curtis N Kollmann TR Levy O Netea MG. Harnessing the beneficial heterologous effects of vaccination. Nat Rev Immunol (2016) 16(6):392–400. doi: 10.1038/nri.2016.43 27157064 PMC4931283

[39] Trunk G Davidovic M Bohlius J. Non-specific effects of bacillus calmette-guerin: a systematic review and meta-analysis of randomized controlled trials. Vaccines (Basel). (2023) 11(1):121. doi: 10.3390/vaccines11010121 PMC9866113 36679966

[40] Wen J Liu Q Tang D He JQ. Efficacy of BCG vaccination against COVID-19: systematic review and meta-analysis of randomized controlled trials. J Clin Med (2023) 12(3):1154. doi: 10.3390/jcm12031154 PMC9917948 36769802

[41] Zapolnik P Kmiecik W Nowakowska A Krzych LJ Szymanski H Stopyra L. A multi-centre, randomised, double-blind, placebo-controlled phase III clinical trial evaluating the impact of BCG re-vaccination on the incidence and severity of SARS-CoV-2 infections among symptomatic healthcare professionals during the COVID-19 pandemic in Poland-evaluation of antibody concentrations. Vaccines (Basel) (2022) 11(1):75. doi: 10.3390/vaccines11010075 PMC9867106 36679920

[42] Bannister S Kim B Dominguez-Andres J Kilic G Ansell BRE Neeland MR. Neonatal BCG vaccination is associated with a long-term DNA methylation signature in circulating monocytes. Sci Adv (2022) 8(31):eabn4002. doi: 10.1126/sciadv.abn4002 35930640 PMC9355358

[43] Freyne B Donath S Germano S Gardiner K Casalaz D Robins-Browne RM. Neonatal BCG vaccination influences cytokine responses to toll-like receptor ligands and heterologous antigens. J Infect Dis (2018) 217(11):1798–808. doi: 10.1093/infdis/jiy069 PMC11491830 29415180

[44] Kleinnijenhuis J Quintin J Preijers F Benn CS Joosten LA Jacobs C. Long-lasting effects of BCG vaccination on both heterologous Th1/Th17 responses and innate trained immunity. J Innate Immun (2014) 6(2):152–8. doi: 10.1159/000355628 PMC3944069 24192057

[45] Kleinnijenhuis J Quintin J Preijers F Joosten LA Ifrim DC Saeed S. Bacille calmette-guerin induces NOD2-dependent nonspecific protection from reinfection *via* epigenetic reprogramming of monocytes. Proc Natl Acad Sci U S A. (2012) 109(43):17537–42. doi: 10.1073/pnas.1202870109 PMC3491454 22988082

[46] Counoupas C Johansen MD Stella AO Nguyen DH Ferguson AL Aggarwal A. A single dose, BCG-adjuvanted COVID-19 vaccine provides sterilising immunity against SARS-CoV-2 infection. NPJ Vaccines (2021) 6(1):143. doi: 10.1038/s41541-021-00406-4 34848711 PMC8633321

[47] Mambelli F Marinho FV Andrade JM de Araujo A Abuna RPF Fabri VMR. Recombinant bacillus calmette-guerin expressing SARS-CoV-2 chimeric protein protects K18-hACE2 mice against viral challenge. J Immunol (2023). 210(12):1925-1937 doi: 10.4049/jimmunol.2200731 PMC10247535 37098890

[48] Brandt L Feino Cunha J Weinreich Olsen A Chilima B Hirsch P Appelberg R. Failure of the mycobacterium bovis BCG vaccine: some species of environmental mycobacteria block multiplication of BCG and induction of protective immunity to tuberculosis. Infect Immun (2002) 70(2):672–8. doi: 10.1128/IAI.70.2.672-678.2002 PMC127715 11796598

[49] Poyntz HC Stylianou E Griffiths KL Marsay L Checkley AM McShane H. Non-tuberculous mycobacteria have diverse effects on BCG efficacy against mycobacterium tuberculosis. Tuberculosis (Edinb). (2014) 94(3):226–37. doi: 10.1016/j.tube.2013.12.006 PMC4066954 24572168

[50] Shah JA Lindestam Arlehamn CS Horne DJ Sette A Hawn TR. Nontuberculous mycobacteria and heterologous immunity to tuberculosis. J Infect Dis (2019) 220(7):1091–8. doi: 10.1093/infdis/jiz285 PMC6736075 31165861

[51] Mangtani P Abubakar I Ariti C Beynon R Pimpin L Fine PE. Protection by BCG vaccine against tuberculosis: a systematic review of randomized controlled trials. Clin Infect Dis (2014) 58(4):470–80. doi: 10.1093/cid/cit790 24336911

[52] Kjaergaard J Birk NM Nissen TN Thostesen LM Pihl GT Benn CS. Nonspecific effect of BCG vaccination at birth on early childhood infections: a randomized, clinical multicenter trial. Pediatr Res (2016) 80(5):681–5. doi: 10.1038/pr.2016.142 27429204

[53] Freyne B Messina NL Donath S Germano S Bonnici R Gardiner K. Neonatal BCG vaccination reduces interferon-γ responsiveness to heterologous pathogens in infants from a randomized controlled trial. J Infect Dis (2020) 221(12):1999–2009. doi: 10.1093/infdis/jiaa030 31990350 PMC7289544

[54] Debisarun PA Kilic G de Bree LCJ Pennings LJ van Ingen J Benn CS. The impact of BCG dose and revaccination on trained immunity. Clin Immunol (2023) 246:109208. doi: 10.1016/j.clim.2022.109208 36565972

[55] Glynn JR Dube A Fielding K Crampin AC Karonga Prevention Trial G Kanjala C. The effect of BCG revaccination on all-cause mortality beyond infancy: 30-year follow-up of a population-based, double-blind, randomised placebo-controlled trial in Malawi. Lancet Infect Dis (2021) 21(11):1590–7. doi: 10.1016/S1473-3099(20)30994-4 PMC8550897 34237262

[56] Roth AE Benn CS Ravn H Rodrigues A Lisse IM Yazdanbakhsh M. Effect of revaccination with BCG in early childhood on mortality: randomised trial in Guinea-Bissau. BMJ (2010) 340:c671. doi: 10.1136/bmj.c671 20231251 PMC2839082

[57] Jolliffe DA Faustini SE Holt H Perdek N Maltby S Talaei M. Determinants of antibody responses to SARS-CoV-2 vaccines: population-based longitudinal study (COVIDENCE UK). Vaccines (Basel) (2022) 10(10):1601. doi: 10.3390/vaccines10101601 PMC9610049 36298466

[58] Kung YA Huang SY Huang CG Liu KT Huang PN Yu KY. Factors influencing neutralizing antibody titers elicited by coronavirus disease 2019 vaccines. Microbes Infect (2023) 25(1-2):105044. doi: 10.1016/j.micinf.2022.105044 36096357 PMC9461341

